# Acute and Chronic Effects of Exercise on Continuous Glucose Monitoring Outcomes in Type 2 Diabetes: A Meta-Analysis

**DOI:** 10.3389/fendo.2020.00495

**Published:** 2020-08-04

**Authors:** Matthew Munan, Camila L. P. Oliveira, Alexis Marcotte-Chénard, Jordan L. Rees, Carla M. Prado, Eléonor Riesco, Normand G. Boulé

**Affiliations:** ^1^Faculty of Kinesiology, Sport, and Recreation, University of Alberta, Edmonton, AB, Canada; ^2^Alberta Diabetes Institute, University of Alberta, Edmonton, AB, Canada; ^3^Faculty of Agricultural, Life & Environmental Sciences, University of Alberta, Edmonton, AB, Canada; ^4^Faculty of Physical Activity Sciences, University of Sherbrooke, Sherbrooke, QC, Canada; ^5^Research Center on Aging, CIUSSS de l'Estrie - CHUS, Sherbrooke, QC, Canada

**Keywords:** exercise, type 2 diabetes, systematic review, meta-analysis, continuous glucose monitoring

## Abstract

**Objective:** To examine the acute and chronic effects of structured exercise on glucose outcomes assessed by continuous glucose monitors in adults with type 2 diabetes.

**Methods:** PubMed, Medline, EMBASE were searched up to January 2020 to identify studies prescribing structured exercise interventions with continuous glucose monitoring outcomes in adults with type 2 diabetes. Randomized controlled trials, crossover trials, and studies with pre- and post-designs were eligible. Short-term studies were defined as having exercise interventions lasting ≤2 weeks. Longer-term studies were defined as >2 weeks.

**Results:** A total of 28 studies were included. Of these, 23 studies were short-term exercise interventions. For all short-term studies, the same participants completed a control condition as well as at least one exercise condition. Compared to the control condition, exercise decreased the primary outcome of mean 24-h glucose concentrations in short-term studies (−0.5 mmol/L, [−0.7, −0.3]; *p* < 0.001). In longer-term studies, mean 24-h glucose was not significantly reduced compared to control (−0.9 mmol/L [−2.2, 0.3], *p* = 0.14) but was reduced compared to pre-exercise values (−0.5 mmol/L, [−0.7 to −0.2] *p* < 0.001). The amount of time spent in hyperglycemia and indices of glycemic variability, but not fasting glucose, also improved following short-term exercise. Among the shorter-term studies, subgroup, and regression analyses suggested that the timing of exercise and sex of participants explained some of the heterogeneity among trials.

**Conclusion:** Both acute and chronic exercise can improve 24-h glucose profiles in adults with type 2 diabetes. The timing of exercise and sex of participants are among the factors that may explain part of the heterogeneity in acute glycemic improvements following exercise.

## Introduction

Meta-analyses have repeatedly confirmed that, on average, regular exercise training causes meaningful improvements in glycemic control in people with type 2 diabetes (T2D) (1, 2). These meta-analyses typically included glycated hemoglobin (A1C) as a primary outcome and showed a high degree of heterogeneity among trials (2). A1C reflects the average glucose concentrations over the last 2–3 months. However, A1C does not provide information on what aspect of glycemic control has been improved (i.e., two people with very different daily glucose profiles can have the same A1C) and does not allow direct comparisons between short-term and longer-term responses to exercise. A better understanding of how exercise affects shorter-term indicators of glycemic control could help better understand how exercise affects longer-term indicators of glycemic control, as well as the heterogenous responses to exercise.

Continuous glucose monitors (CGM) can measure interstitial glucose concentrations at frequent intervals over several days. In addition to mean daily glucose concentration, CGM permit measures such as glucose concentrations over specific periods (e.g., post-prandial periods), the amount of time within specific glucose ranges (e.g., below 3.9 mmol/L), or other outcomes such as glucose variability, which can be associated with oxidative stress (3) and potentially other diabetes-related complications (4).

In 2013, members of our team published the first meta-analysis on the effects of exercise on CGM outcomes based on eight short-term studies and three longer-term studies (5). Synthesis of results from short-term studies revealed that exercise reduced outcomes such as mean 24-h glucose and time spent in hyperglycemia but did not affect other outcomes such as fasting glucose. Due to the low number of studies, we had limited our subgroup comparisons to aerobic vs. resistance exercise. Since then, the number of exercise and CGM studies in T2D has increased rapidly.

Therefore, the purpose of this meta-analysis was to provide an updated systematic review of the effects of exercise on CGM outcomes in T2D. Given the heterogeneity identified in previous meta-analyses, we explored differences among the short-term trials with pre-specified and novel subgroup comparisons, as well as meta-regression analyses, to examine the impact of factors such as exercise timing, dietary standardization, medications, type of CGM, sex, and baseline glycemic control.

## Methods

### Search Strategy

On January 9 of 2020, a literature search of EMBASE, PubMed, and Medline were performed using terms relating to exercise, T2D and CGM. Search results were combined into a bibliographic software (Endnotes X6, Thomson Reuters, Toronto, Canada) and duplicates were eliminated using an automated feature. Details of the literature search strategy are available in Supplementary Table 1.

Two reviewers independently read titles and abstracts. Any record that was deemed to meet the inclusion criteria was selected for a full-text review (i.e., agreement between reviewers was not required at this stage). Two reviewers then reviewed all selected full-text articles for eligibility and any disagreement was resolved through discussion with a third reviewer.

### Study Selection

Eligibility was determined according to the following inclusion criteria:

- **Population:** Only studies with data from adults with T2D were eligible. Studies were not eligible if data were combined for people with and without diabetes, or with people above and below 18 years of age.
- **Intervention:** Both short-term (i.e., ≤2 weeks) and longer-term studies (>2 weeks) were included if they examined the effects of structured exercise interventions defined in terms of frequency, intensity, type, and duration. Interventions that encouraged participants to become more active without providing structured prescriptions or monitoring (e.g., direct supervision or logs) were not eligible. Since developing this criterion for our previous meta-analysis (5) several studies examined the effects of breaking up sedentary time with exercise. These studies were not included to facilitate comparisons with our previous meta-analysis and because they often involved restricting activities during the control condition (e.g., prolonged sitting). In such studies, it was unclear if differences between conditions were due to the activity itself or the impact of prolonged sitting in the control condition.
- **Comparison:** A non-exercise control condition was required for comparison to the exercise condition. Both randomized and non-randomized (e.g., pre vs. post) comparisons were eligible, as were trials that employed parallel or crossover designs. Studies comparing combined exercise and dietary interventions to a control condition not receiving the dietary intervention were not eligible.
- **Outcome:** Studies were required to provide data from CGM or “Flash” glucose monitoring over a day (i.e., approximately 24 h) from both the exercise and control conditions. Mean 24-h glucose was considered the primary outcome of interest.

### Data Extraction

Two reviewers extracted the following CGM outcomes in duplicate: mean 24-h glucose, time in hyperglycemia, time in hypoglycemia, time in range, post-prandial glucose, fasting glucose, nocturnal glucose, and glucose variability. Recent international consensus statements (6) suggest values of 3.9–10.0 mmol/L for time in range, but we also extracted data from articles who had similar definitions but slightly different cutoffs (e.g., 4.0 instead of 3.9 mmol/L, or 9.0 instead of 10.0 mmol/L). Indicators of glucose variability included mean amplitude of glucose excursions (MAGE), continuous overall net glycemic action (CONGA), or standard deviation (SD). Participant characteristics and details of the interventions were extracted by a single reviewer and verified by a second reviewer. Participant characteristics included age, sex, body mass index (BMI), duration of diabetes, menopausal status, the type of CGM, and type of glucose lowering medication they were treated with, and A1C. Characteristics of the exercise intervention included the type of exercise, the frequency and duration of exercise sessions, as well as the intensity. We noted if meals were provided as a means of standardizing diet between the exercise and control conditions and categorized groups into: all meals provided, meals partially provided, or no meals provided. The timing of exercise in relation to meals was categorized as fasting, after breakfast, afternoon (i.e., before dinner), or evening (i.e., after dinner).

Several data transformations were made before combining data from trials. Glucose concentrations in mg/dL were converted and presented as mmol/L by dividing by 18. Since CGM measures are provided in constant time intervals (e.g., 5 min), the area under the curve data was converted to mean glucose by dividing the total area by the amount of time. The percent time in hyper- or hypoglycemia was transformed into minutes by multiplying the percentage by the total amount of time.

Based on our previous meta-analysis (5), we expected participants in the short-term studies to complete both the exercise and control conditions (e.g., crossover trials) even if some would not be in randomized order. The primary analyses for these studies were based on the within-person difference in glucose concentrations. In instances where the SD or standard error (SE) of the change was not reported, it was estimated from *p*-values as described in section 7.7.3.3 of the Cochrane Handbook (7). In cases where information was displayed in a figure, mean difference and SD was estimated using plot digitizer software (Plot Digitizer Version 2.1 ©Joseph Huwaldt). In infrequent cases, we were unable to estimate the SE of the change from any of the above methods. In such cases, we used the correlation coefficient between exercise and control values that we calculated from other studies to estimate the SE of the change as described in section 16.1.3.2 of the Cochrane Handbook (7).

### Risk of Bias

Two authors independently performed risk of bias assessment. Risk of bias was assessed using a domain-based evaluation, in which seven specific domains were addressed: (1) sequence generation, (2) allocation concealment, (3) blinding of participants and personnel, (4) blinding of outcome assessment, (5) incomplete outcome data, (6) selective outcome reporting, and (7) other bias. A judgement of “low risk,” “high risk,” and “unclear risk” of bias was assigned for each study, according to the criteria in the Cochrane Collaboration's Risk of Bias Tool; section 8.5 in the Cochrane Handbook (7). These criteria had been updated since our previous review (5). For example, describing a trial as randomized was no longer sufficient to be categorized as “low risk” for “sequence generation”; the authors were required to describe an appropriate method for randomization.

### Statistical Analysis

Statistical analyses were performed using Review Manager Software (Revman 5.3, Cochrane Collaboration, Copenhagen Denmark). For all shorter-term studies, participants completed both conditions (crossover trials or pre- and post-designs). For these trials, the mean difference (MD) and the within participant SE of this difference were pooled using the generic inverse variance method to calculate a weighted mean difference (WMD).

For the longer-term trials that randomly assigned participants to either exercise vs. control conditions, the primary analyses considered mean differences between conditions which was pooled using a random effects model. When a control condition was compared to multiple exercise conditions, the sample size of the control condition was divided by the number of comparisons. Three of the five longer-term trials did not include a control condition. Therefore, secondary analysis compared pre- vs. post-exercise data from all longer-term trials using the generic inverse variance method.

Heterogeneity was examined through the chi-square test and also presented using the *I*^2^ statistic, which describes the percentage of the variability that is due to heterogeneity rather than chance (7). When the *I*^2^ was above 40%, heterogeneity was explored with subgroup and meta-regression analyses. As in previous meta-analyses (5, 8), subgroups were pre-defined according to type of exercise (i.e., aerobic, vs. high-intensity interval training, vs. resistance). As suggested in the study by Rees et al. (9), other factors such as exercise timing, and dietary intervention may have influenced the results and were therefore included in subgroup analyses. Lastly factors such as the type of CGM (real time vs. blinded vs. intermittently scanned) and the type of glucose lowering medications taken by participants were added during the review process. Meta-regression analyses included the proportion of participants who were female, A1C, and glucose concentrations from the control condition as predictors. For all the short-term studies, the same participants completed the control and exercise conditions.

## Results

### Description of Studies

**Figure 1:**
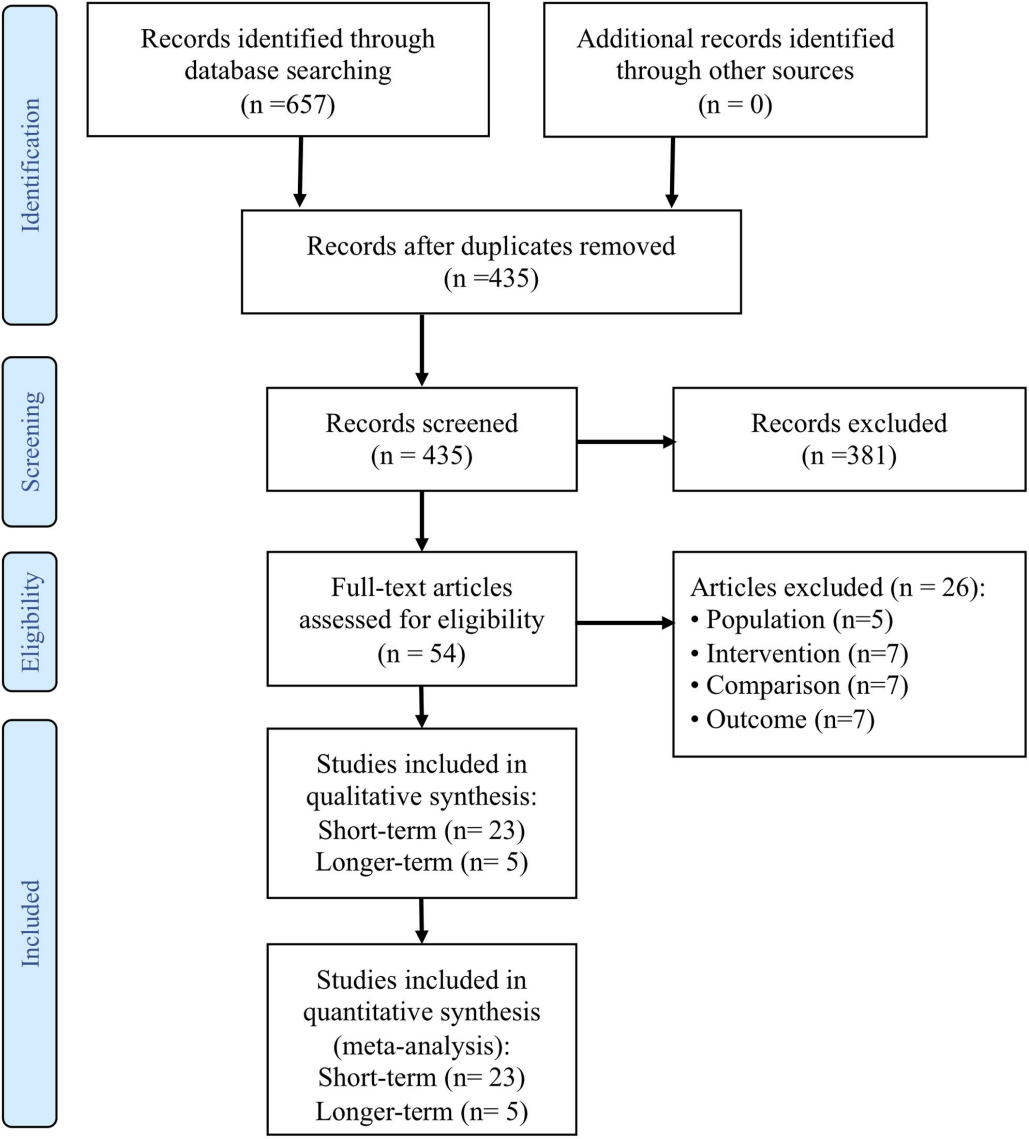
PRISMA study flow diagram.

The literature search retrieved 657 records (see PRISMA Trial Flow diagram in Figure 1). After duplicates were removed, 435 records were reviewed. Fifty-four full text articles were screened and 26 were excluded for the following reasons:

- **Population**. Studies that were not exclusively conducted in adults with T2D (10–14) were excluded. Of these, the study by Newton and White (12) was included in our first meta-analyses, but excluded this time because the age range was from 14 to 20 years old.
- **Intervention**. Studies were excluded when they had co-interventions, such as changes in medication or insulin (15, 16), which influenced the changes caused by exercise. There was also one study in three records (17–19) examining the effect of Yoga, but it was excluded since we were unable to extract sufficient detail on the structure of exercise component, or control for any effect of the breathing exercise or meditation components of the intervention. Two studies examined the effect of breaking up sedentary time (20, 21) with several short bouts of activity. The control condition in these studies involved restricting movement by sitting from 8 to 14 h (20, 21). It therefore became difficult to know how much of the difference between the activity and control conditions was due to the physical activity itself or the prolonged sedentary behavior, which was likely greater than in free-living conditions. The study by Blankenship et al. (22) included a continuous walking condition and another activity condition with 12 breaks in sedentary time. However, the control condition asked participants to maintain their habitual physical activity behavior and we therefore chose to include the control vs. walking comparison.
- **Comparison**. Studies that did not include a non-exercise control condition were excluded (23–29). Of these trials, the one by Bacchi et al. (25) had been included in the qualitative synthesis of our 2013 systematic review. We excluded it in the present analysis because the control condition started 24 h after the exercise condition and we could not rule out that the effect of exercise did not persist beyond 24 h. In the study by Godkin et al. (30), the effect of a single bout of exercise was compared to control after the first session of exercise and after a session of exercise performed after 6 weeks of exercise training. We only included the effects of the first session of exercise since this was more comparable to the other included studies.
- **Outcomes**. Some studies did not have usable CGM data (31, 32) or presented data which was available from the same population as another included study (33– 37). For example, Little et al. (34) included the same participants as the study by Gillen et al. (38). These articles were different in that Gillen et al. examined participants after one bout of exercise while Little et al. examined participants after six bouts of exercise. Another difference was that Little et al. assessed glycemic control starting ~48 h after the last training bout; a period that was inconsistent with the rest of the short-term studies. To favor homogeneity among studies only the results from Gillen et al. was included. The study from Savikj et al. (39) provided data from week 1 and week 2 of training but we only included the data from week 1.

Table 1 includes characteristics of the 23 eligible short-term studies. A total of 373 participants were included. The majority of these participants were males (264 males vs. 109 females). Many of the studies included multiple exercise groups for a total of 40 exercise groups. There were a variety of exercise prescriptions, with studies prescribing low, moderate, and high-intensity aerobic exercise, including different forms of high-intensity interval training (HIIT). The timing in relation to meals varied among studies but was reported in all but 2 studies. Eleven studies provided all of the meals to the participants throughout the 24-h period, 6 studies provided some meals but not all, and 6 studies did not provide any meals. In the studies that did not provide meals, or partially provided meals, participants were often asked to maintain similar dietary intakes across conditions. Of the 23 short-term studies, one study used an intermittently scanned CGM (Freestyle Libre, Abbott). Three studies used the Guardian or MiniMed (Medtronic) CGM which provided real-time data to participants. Five studies used GlucoDay S (A. Menarini Diagnostics) CGM, which has the capability of showing real time glucose concentrations but was likely blinded. An additional 12 studies used iPro (Medtronic) CGM technology, which are blinded to participants and researchers until the data is download after removal of the sensor. An additional three studies did not specify the type of Medtronic CGM but provided enough detail to suggest that the data were also examined retrospective and not available in real-time.

**Table 1:** Characteristics of included short-term (≤2 weeks) studies.

**Source**	**(*N*) M/F**	**Age (yr)**	**BMI (kg/m^**2**^)**	**Duration T2D (yr)**	**A1C (%)**	**Type of exercise**	**Exercise intensity**	**Exercise duration**	**Timing of exercise**	**Meals during CGM**
1. Blankenship et al. (22)^*^	(30) 14/16	64 ± 8.2	31.7 ± 5.4	10.0 ± 7.8	7.4 ± 1.1	- Walking	- “Faster than usual walking speed”	−1 bout (20, 40, or 60 min)	- Morning (30–60 min post-breakfast)	Partially provided
2. Cruz et al. (40)	(12) 0/12	55.2 ± 4.0	29.0 ± 5.4	5.7 ± 3.7	NR	- Resistance - Resistance	−40% 1RM - 80% 1RM	−1 bout (40 min) - 1 bout (40 min)	- Morning - Morning	Partially provided
3. Erickson et al. (41)	(8) 5/3	60 ± 10.7	33.8 ± 10.3	NR	7.9 ± 2.3	- Walking	−50% V0_2_ Peak	1 bout (3 × 10 min)	- Morning	All provided
4. Figueira et al. (42)	(14) 5/9	56 ± 7	30 ± 4	4.5 [3.1–5.9]	7.9 ± 2.6	- Cycling - Cycling and Resistance	−70% Peak HR - 70% Peak HR and 4 exercises 65% 1RM	−1 bout (40 min) - 1 bout (20 min) and 3 sets of 12 reps	- Morning -Morning	None provided
5. Gillen et al. (38)	(7) 4/3	62 ± 3	30.5 ± 1.9	>3 month	6.9 ± 0.7	- Cycling	−85% max HR	−1 bout, 10 × 60 s intervals (10 min)	- Morning	All Provided
6. Godkin et al. (30)	(7) 5/2	21 to 70	31 ± 5	6 ± 9	6.5 ± 0.7	- Stair climbing	- HIIT: Mean HR = 74 ± 5% of max HR	−1 bout: 3 × 1:1 min stairs: walking	- Morning	All provided
7. Haxhi et al. (43)	(9) 9/0	52.8 ± 6.6	30.2 ± 3.1	5.2 ± 4.3	7.0 ± 0.6	- Walking -Walking	−50% HRR - 50% HRR	−2 bouts (20 min) - 1 bout (40 min)	- Split before and after lunch - Afternoon	Partially provided
8. Karstoft et al. (44)	(10) 7/3	60.3 ± 2.3	28.3 ± 1.1	6 ± 0.9	6.3 ± 0.6	- Walking -Walking	- Interval @ 54–89% VO_2peak_ (3:3 min) - 73% VO_2peak_	−1 bout (60 min) - 1 bout (60 min)	- Fasting - Fasting	None provided
9. Karstoft et al. (45)	(14) 11/3	65 ± 2	18 to 39.9	9 ± 1	6.6 ± 1.1	- Walking -Walking	- Interval @ 54–89% VO_2peak_ (3:3 min) - 73%VO_2peak_	−10 bouts (60 min) - 10 bouts (60 min)	- Not specified - Not specified	Partially provided
10. Li et al. (46)	(29) 22/7	51.0 ± 11.2	24.8 ± 3.4	5.7 ± 3.4	7.3 ± 1.3	- Walking	−40% HRR	−1 bout (20 min)	- Evening (post-dinner)	All provided
11. Macdonald et al. (47)	(6) 5/1	59 ± 3	32 ± 1.4	2.0 ± 0.5	8.4 ± 1.7	- Cycling	−90% LT	−1 bout (60 min)	- Fasted (morning)	None provided
12. Manders et al. (48)	(9) 9/0	57 ± 6	29 ± 3.0	9 ± 12	7.1 ± 1.2	- Cycling -Cycling	−35% Wmax - 70% Wmax	−1 bout (60 min) - 1 bout (30 min)	- Morning (1 h post-breakfast)	All provided
13. Metcalfe et al. (49)	(11) 11/0	52 ± 6	29.7 ± 3.1	4 ± 3	7.0 ± 0.8	- Cycling - Cycling -Cycling	- REHIT-all out - HIIT-85% Wmax - MICT-50% Wmax	−1 bout (10 min) - HIIT (10 min) - MICT (30 min)	-Morning (30 min post-breakfast)	All provided
14. Mikus et al. (50)	(13) 8/5	53.0 ± 7.2	34.1 ± 4.7	NR	6.6 ± 0.6	- Alternating walk/cycle	−60–75% HRR	−7 days (60 min/day)	- Not specified	None provided
15. Myette-Côté et al. (51)	(10) 5/5	59 ± 9.6	29.5 ± 4.7	7.5 ± 5.2	6.6 ± 0.6	- Walking	−85% VT	−1 bout (50 min)	- Morning	Partially provided
16. Oberlin et al. (52)	(9) 5/4	60.3 ± 3	36.0 ± 1.1	NR	6.3 ± 0.6	- Alternating walk/cycle	−60% HRR	−1 bout (60 min) - (20:20:20 min of walk:cycle:walk)	- Fasting	All provided
17. Praet et al. (53)	(11) 11/0	59.1 ± 7.6	32.2 ± 4.0	12.1 ± 7.0	7.6 ± 1.0	- HIIT and Resistance	- HIIT: 30:60 s @ 50%Wmax:15 W; - Resistance: 2 sets ×10 reps @ 50% 1RM	−1 bout (45 min)	- Morning	None provided
18. Rees et al. (9)	(63) 29/34	64.4 ± 8.0	30.5 ± 6.5	9.7 ± 6.1	6.8 ± 0.7	- Walking	−5.0 km/h, 0.5% incline	−1 bout (50 min)	- Afternoon	All provided
19. Savikj et al. (39)	(11) 11/0	60 ± 7	27.5 ± 2.0	11 ± 10	6.6 ± 1.3	- HIIT cycling - HIIT cycling	−180–350 W - 180–350 W	−6 × 60:60 s intervals - 6 × 60:60 s intervals	- Morning, with snack available - Afternoon	None provided
20. Terada et al. (54)	(10) 8/2	60 ± 6	30.8 ± 5.4	6.8 ± 4.6	7.1 ± 1.0	- Walking - HIIT Walk - Walking - HIIT Walk	−55%VO_2peak_ - 3:1 min @ 40:100% - 55%VO_peak_ - 3:1 min 40–100%	−1 bout (60 min) - 1 bout (60 min) - 1 bout (60 min) - 1 bout (60 min)	- Fasting - Fasting - Morning -Morning	Partially provided
21. Van Dijk et al. (55)	(15) 15/0 (15) 15/0	Insulin 61 ± 4 No-Ins 60 ± 4	Insulin 29.7 ± 4.3 No-Ins 29.7 ± 3.5	Insulin 13.5 ± 8.5 No-Ins 6.5 ± 3.9	Insulin 7.6 ± 1.2 No-Ins 7.5 ± 0.8	- Cycling - Resistance - Cycling -Resistance	−50% Wmax - 5 sets of 10 reps (55–75% 1RM) - 50% Wmax - 5 sets of 10 reps (55–75% 1RM)	−1 bout (45 min) - 1 bout (45 min) - 1 bout (45 min) - 1 bout (45 min)	- Morning - Morning - Morning -Morning	All provided
22. Van Dijk et al. (56)	(30) 30/0	60 ± 6	31.1 ± 3.8	8.1	7.2 ± 1.1	- Cycling -Cycling	−50% Wmax - 50% Wmax	−2 bouts (30 min on 2 days) - 1 bout (60 min)	- Morning -Morning	All provided
23. Van Dijk et al. (57)	(20) 20/0	61 ± 4	29.5 ± 4.0	8 ± 4	6.9 ± 0.4	- Cycling - Walking (“strolling”)	- ~6.0 METS - ~3.0 METS	−1 day (1 × 45 min) - 1 day (3 × 15 min)	- Morning - After Each Meal	All provided

*Data presented as mean ± standard deviation, n, sample size; M, males; F, females; yr, years; A1C, glycated hemoglobin; BMI, body mass index; T2D, type 2 diabetes; METS, metabolic equivalent; V0_2_, oxygen consumption; NR, not reported; HR, heart rate; HRR, heart rate Reserve; VT, ventilatory threshold; LT, lactate threshold; Wmax, peak workload; REHIT, reduced exertion high intensity interval training; HIIT, high intensity interval training; MICT, moderate-intensity continuous training*.

* *The Blankenship et al. (22) study also included a condition with breaking sedentary time, which was not included in the meta-analysis*.

Twenty of the 23 short-term studies provided some information on the type of medication. Of the 373 participants from the 23 short-term trials, we were able to determine that the most common medications were: metformin (taken by at least 70% of participants), sulfonylureas (taken by at least 17% of participants), insulin (taken by at least 11% of participants), and DPP4 inhibitors (taken by at least 10% of participants). Other classes of medications were each taken by ≤5% of the participants. Menopausal status was reported in 6 of the 15 short-term studies that included women. In these 6 studies, almost all participants were postmenopausal (a total of only 3 women were not).

Table 2 describes the five eligible longer-term studies. Interventions ranged from 8 to 16 weeks in duration. A total of 99 participants (57 males and 42 females) were included in 9 different exercise interventions, but only 15 participants in two separate control groups (60, 62). Francois et al. (59) had three separate groups performing the same HIIT training protocol, but we only included one of these groups in our analyses because the others received a skimmed-milk supplement or a macronutrient matched control beverage, making it unclear what effects were due to the exercise or supplements. Consequently, we only included the HIIT group that received the flavored water placebo from Francois et al. (59). Of the five longer-term studies, two used the blinded iPro CGM, two used the Guardian CGM and one used a MiniMed system that also included a portable monitor (all from Medtronic).

**Table 2:** Characteristics of longer-term (>2 weeks) exercise studies.

**Source**	**Group**	**(*N*) M/F**	**Age (yr)**	**BMI (kg/m^**2**^)**	**Duration T2D (yr)**	**A1C (%)**	**Duration of intervention**	**Frequency exercise**	**Length of exercise**	**Intensity of exercise**	**Meals during CGM**
1. Cauza et al. (58)	Resistance Endurance	(8) 3/5 (7) 1/6	55.1 ± 4.8 60.3 ± 8.2	29.9 ± 2.3 36.3 ± 12.4	9 ± 11 9 ± 11	7.5 ± 1.4 8.0 ± 1.1	16 weeks 16 weeks	3/week 3/week	10 exercise 15–30 min	1–2 sets @ 10–15 reps 60% VO_2max_	None provided
2. Francois et al. (59)^*^	HIIT (with aerobic and resistance)	(19) 8/11	55 ± 9	33 ± 6	5 ± 6	6.9 ± 0.8	12 weeks	3/week: 2 aerobic, 1 resistance	20 min (1:1 min intervals)	Aerobic: 90%HRmax Resistance: RPE 5/10	None provided
3. Karstoft et al. (60)	Control Walking Interval Walking	(8) 5/3 (12) 8/4 (12) 7/5	57.1 ± 8.5 60.8 ± 7.6 57.5 ± 8.3	29.7 ± 5.4 29.9 ± 5.5 29.0 ± 4.5	4.5 ± 4.2 6.2 ± 5.2 3.5 ± 2.4	6.4 ± 0.6 6.6 ± 0.7 6.9 ± 0.7	16 weeks 16 weeks 16 weeks	NA 5/week 5/week	NA 60 min 60 min (3:3 min intervals)	NA 55% of peek EE 70:40% peek EE	None provided
4. Ruffino et al. (61)	Walking REHIT	(16) 16/0	55 ± 5	30.6 ± 2.8	4 ± 4	NR	8 weeks 8 weeks	3/week 5/week	30 min 10 min	40–55% of HRR Cycling @ 25 Watt +2 sprints of 10–20 s @ 0.65 Nm/kg lean mass	All provided
5. Winding et al. (62)	Control Endurance HIIT	(7) 5/2 (12) 7/5 (13) 7/6	57 ± 7 58 ± 8 54 ± 6	28.0 ± 3.5 27.4 ± 3.1 28.1 ± 3.5	7 ± 5 6 ± 4 8 ± 4	7.0 ± 1.2 6.6 ± 0.9 6.8 ± 0.8	11 weeks 11 weeks 11 weeks	NA 3/week 3/week	NA 40 min 20 min (1:1 min intervals)	NA 50% Wpeak 95:20% Wpeak	None provided

*Data presented as mean ± standard deviation, n, sample size; M, males; F, females; yr, years; A1C, glycated hemoglobin; BMI, body mass index; T2D, type 2 diabetes; min, minutes; EE, energy expenditure; HIIT, high-intensity interval training; REHIT, reduced exertion high intensity interval training; HRmax, maximum heart rate; HRR, heart rate reserve; CGM, continuous glucose monitoring; NR, not reported; RPE, rating of perceived exertion; VO_2_max, maximal oxygen consumption; Wpeak, peak workload. Some participants in the control group we randomized to one of the exercise groups in the Karstoft et al. (60) and Winding et al. (62) studies*.

* *Francois et al. (59) also had two other HIIT groups with dietary interventions that are not included in the meta-analysis*.

### Effect of Short-Term Exercise ( ≤ 2 Weeks) on Glucose Concentrations

**Figure 2:**
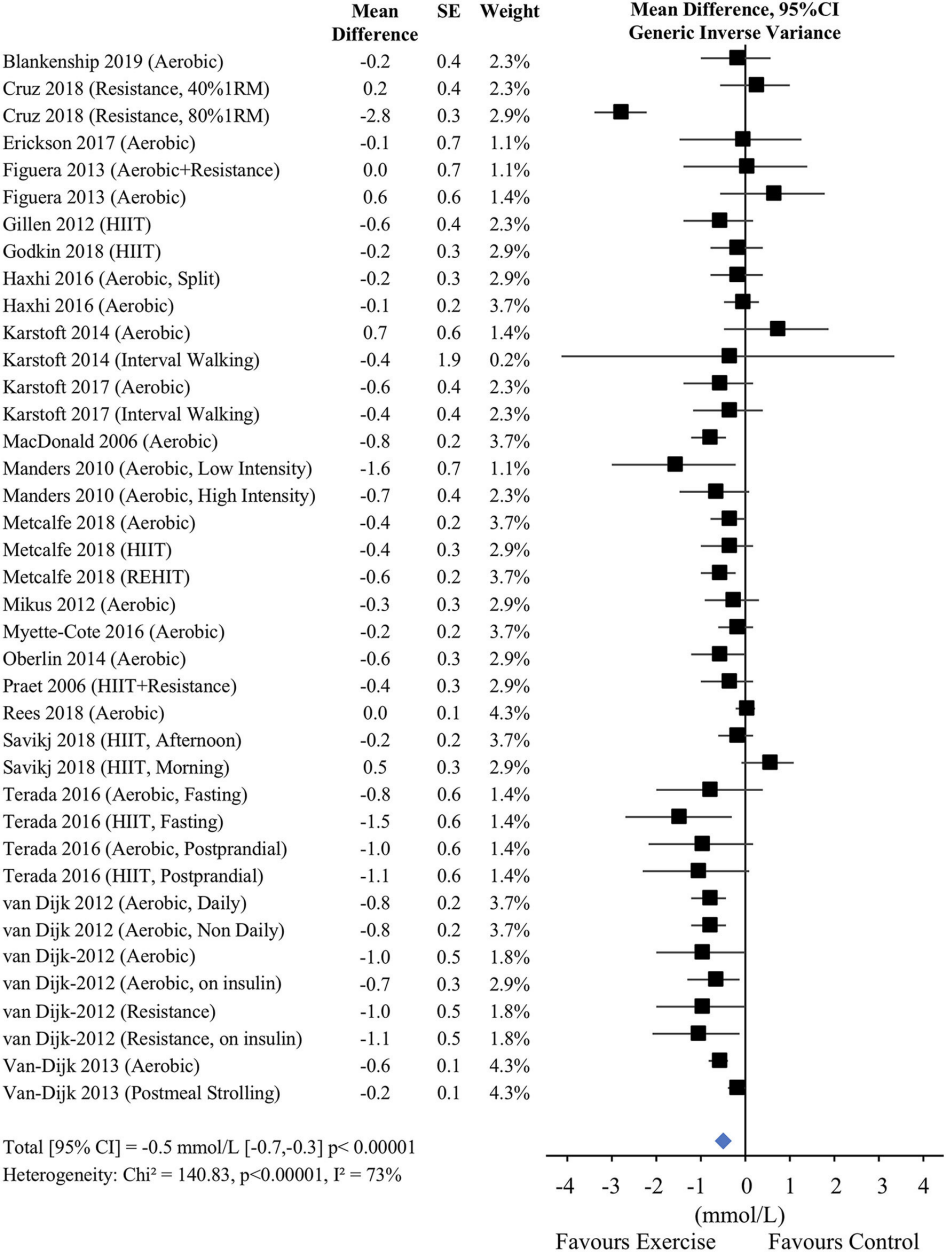
Mean 24-h glucose concentrations in short-term (≤2 weeks) studies. CI, confidence interval; SE, standard error; 1RM, one repetition maximum; HIIT, high-intensity interval training; REHIT, reduced exertion high intensity interval training.

Among the 23 short-term studies, 22 reported 24-h glucose concentrations. Several studies had multiple exercise conditions, which led to a total of 39 exercise groups included in the overall analyses. Compared to control, exercise reduced 24-h glucose concentrations by 0.5 mmol/L, 95% CI [−0.7 to −0.3] (*p* < 0.001, see complete details in Figure 2). However, there was a high degree of heterogeneity among trials (Chi^2^ = 140.8, *p* < 0.001); *I*^2^ = 73%). This heterogeneity was only partially reduced (Chi^2^ = 76.1, *p* < 0.001, I^2^ = 51%) after removing a visual outlier [i.e., the group performing resistance training at 40% of their 1-repetition maximum from Cruz et al. (40)].

Due to the significant heterogeneity among studies, analysis was performed by dividing studies into subgroups according to the timing of exercise, type of exercise, dietary control, and type of CGM (see Table 3). Of these subgroups, only the exercise timing analyses identified heterogeneity among subgroups (*p* < 0.001). There were significant reductions in mean 24-h glucose when exercise was performed in the fasted state (−0.7 mmol/L [−1.1, −0.2], *p* = 0.004) and in the morning (−0.6 mmol/L [−0.9, −0.4], *p* < 0.001) but not in the afternoon (−0.1 mmol/L [−0.2, 0.1], *p* = 0.54). Heterogeneity remained elevated in the morning subgroup but was reduced from *I*^2^ = 75% to *I*^2^ = 38% when the outlier from Cruz et al. (40) was removed.

**Table 3:** Subgroup analyses for changes in mean 24-h glucose in short-term (≤2 weeks) studies.

**Subgroup**	**Number of subgroups**	**Effect estimate**	**Heterogeneity**
**Overall**	39	−0.5 [−0.7, −0.3], *p* < 0.001	Chi^2^ = 140.8, *p* < 0.001, *I*^2^ = 73%
**Exercise timing** Fasting Morning Afternoon None of the above^*^	6 24 3 6	−0.7 [−1.1, −0.2], *p* = 0.004 −0.6 [−0.9, −0.4], *p* < 0.001 −0.1 [−0.2, 0.1], *p* = 0.54 −0.2 [−0.4, −0.1], *p* = 0.005 **Subgroup differences:** ***p*** **<** **0.001**	Chi^2^ = 7.6, *p* = 0.18, *I*^2^ = 35% Chi^2^ = 91.0, *p* < 0.00001 I^2^ = 75% Chi^2^ = 0.9, *p* = 0.65, *I*^2^ = 0% Chi^2^ = 1.2, *p* = 0.94, *I*^2^ = 0%
**Exercise type** Continuous aerobic HIIT/REHIT Resistance Aerobic and resistance	24 9 4 2	−0.4 [−0.6, −0.3], *p* < 0.001 −0.4 [−0.7, −0.1], *p* < 0.02 −1.2 [−2.6, 0.3], *p* = 0.11 −0.3, [−0.9, 0.2], *p* = 0.22 **Subgroup differences:** ***p*** = **0.76**	Chi^2^ = 54.2, *p* < 0.001, *I*^2^ = 58% Chi^2^ = 15.8, *p* = 0.05, *I*^2^ = 49% Chi^2^ = 38.5, *p* < 0.001, *I*^2^ = 92% Chi^2^ = 0.28, *p* = 0.60, *I*^2^ = 0%
**Dietary control** No meals provided Meals partially provided All meals provided	10 11 18	−0.2 [−0.5, 0.2], *p* = 0.29 −0.7 [−1.3, −0.1], *p* = 0.01 −0.5 [−0.7, −0.3], *p* < 0.001 **Subgroup differences:** ***p*** = **0.16**	Chi^2^ = 19.5, *p* < 0.02, *I*^2^ = 54% Chi^2^ = 74.6, *p* < 0.001, *I*^2^ = 87% Chi^2^ = 43.7, *p* < 0.001, *I*^2^ = 61%
**Type of CGM** Real-time Blinded Intermittently scanned	6 31 2	−0.3 [−1.9, 1.3], *p* = 0.70 −0.5 [−0.6, −0.4], *p* < 0.001 [−0.6, 0.8], *p* = 0.74 **Subgroup differences:** ***p*** = **0.23**	Chi^2^ = 62.2, *p* < 0.001, *I*^2^ = 92% Chi^2^ = 58.1, *p* = 0.002, *I*^2^ = 48% Chi^2^ = 3.8, *p* = 0.05, *I*^2^ = 73%
**Randomization** Low or unclear risk High risk	33 6	−0.5 [−0.6, −0.4], *p* < 0.001 −0.4 [−0.9, 0.1], *p* < 0.001 **Subgroup differences:** ***p*** = **0.71**	Chi^2^ = 56.4, *p* < 0.001, *I*^2^ = 54% Chi^2^ = 83.3, *p* < 0.001, *I*^2^ = 87%

*Analyses performed according to the generic inverse variance method. From the 22 included studies, several had multiple exercise interventions for a total of 39 subgroups. HIIT, high-intensity interval training; REHIT, reduced exertion high intensity interval training; CGM, continuous glucose monitor*.

* *The difference among exercise timing subgroup remained after removing the “none of the above” subgroup, which included exercise interventions for which the timing was not specified or split over different times of the day*.

**Figure 3:**
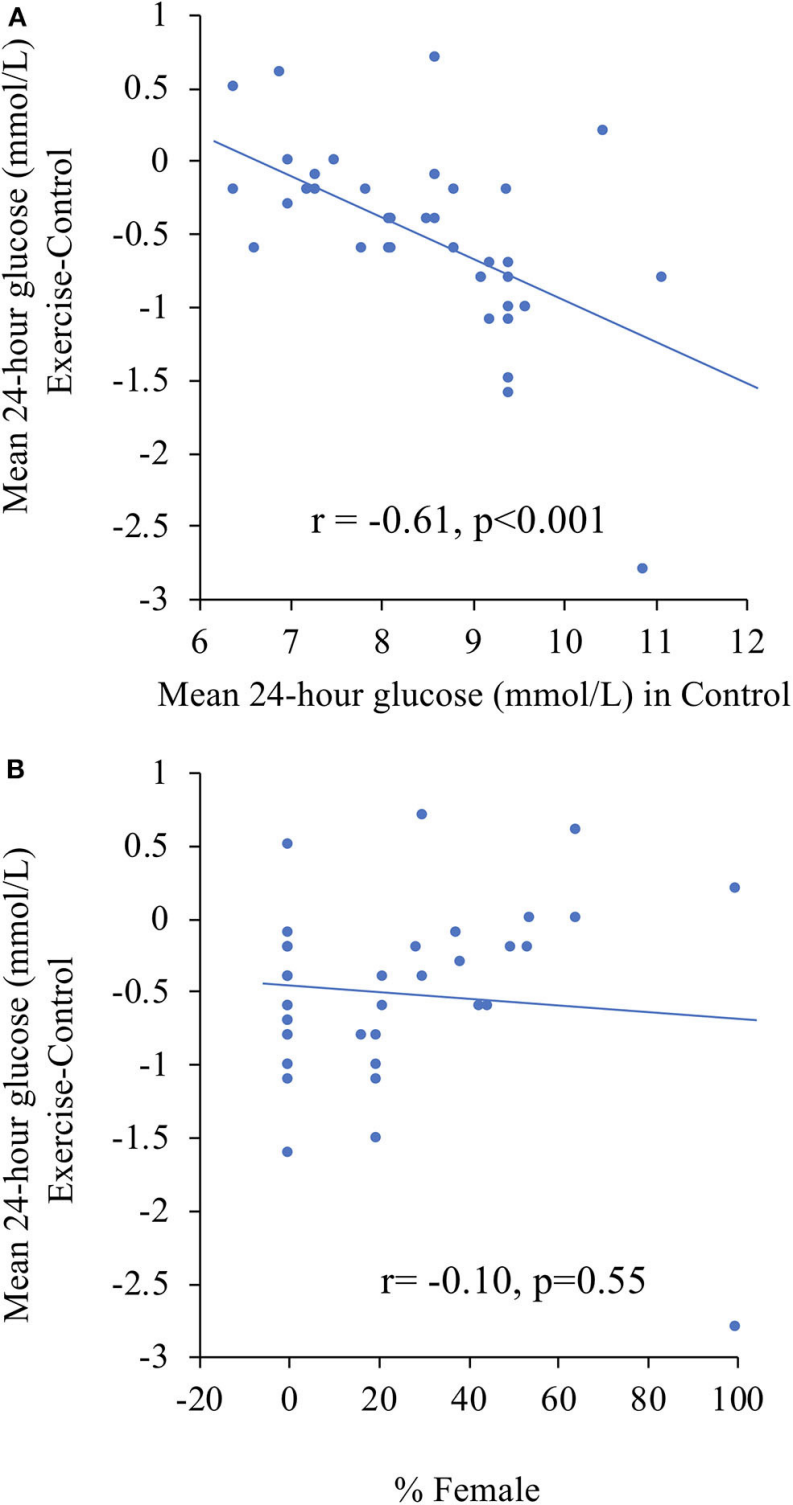
Meta-regression to predict changes in mean 24-h glucose concentrations following exercise according to: **(A)** mean 24-h glucose concentrations in the control condition, and **(B)** percentage of females. The correlation coefficients were changed to *r* = −0.53 (*p* < 0.001) and *r* = 0.39 (*p* = 0.016), respectively, after removing the potential outlier with the largest decrease in mean 24-h glucose.

Meta-regression was performed to predict changes in 24-h glucose concentrations following exercise with other variables such as 24-h glucose concentrations in the control condition, baseline A1C, age, BMI, or the percentage of female participants. Greater mean 24-h glucose concentration in the control condition predicted a greater decrease in 24-h glucose concentrations following exercise (*r* = −0.61, *p* < 0.001), as shown in Figure 3. Note that the same participants completed both the control and exercise conditions (i.e., repeated measures). When mean 24-h glucose from the control condition was replaced by A1C as an indicator of glycemic control, the relationship was in the same direction (*r* = −0.33, *p* = 0.04). The proportion of females within a study was not associated with improvements in 24-h glucose (*r* = −0.10, *p* = 0.55), but when the aforementioned outlier was removed the correlation became positive and statistically significant (*r* = 0.39, *p* = 0.016) suggesting that studies with a greater proportion of females observed smaller improvements in mean 24-h glucose.

A greater proportion of participants treated with sulfonylureas within a study was associated with greater reductions in mean 24-h glucose following exercise (*r* = −0.34, *p* = 0.04). Use of other medications, including metformin (*r* = 0.20, *p* = 0.25), were not significantly associated with changes in 24-h glucose concentrations. Other variables such as exercise duration, age and BMI were not associated with changes in 24-h glucose concentration when examined among all studies or only among studies prescribing continuous aerobic exercise (all *p* > 0.30).

Change in secondary glycemic outcomes are summarized in Table 4. Time spent in hyperglycemia was analyzed from 16 studies, which included 30 exercise vs. control comparisons. There was a significant reduction in the daily time spent in hyperglycemia (−94 min [−115, −72], *I*^2^ = 53%). The subgroup differences reflected the findings from the 24-h glucose concentrations but are not presented as some of the subgroups were much smaller (e.g., only a single study). Indices of glycemic variability were reported in 11 studies with a total of 18 subgroups. Many different measures (e.g., MAGE, SD, and CONGA) were reported in the individual studies. MAGE was the most frequently reported index of glycemia variability and was available in all but two subgroups. MAGE was reduced by −0.41 [−0.63, −0.20] (Chi^2^ = 19.65, *p* = 0.19; *I*^2^ = 24%). On the other hand, fasting glucose and time in hypoglycemia were not significantly affected by exercise.

**Table 4:** Analyses of secondary outcomes.

**Outcome**	**Number of subgroups**	**Effect estimate**	**Heterogeneity**
Time in hyperglycemia (min)	30	−94 [−115, −72], *p* < 0.001	Chi^2^ = 61.73, *p* = 0.0004, *I*^2^ = 53%
Time in hypoglycemia (min)	12	−2 [−11, 7], *p* = 0.67	Chi^2^ = 14.81, *p* = 0.19, *I*^2^ = 26%
Glycemic variability (MAGE)	16	−0.41 [−0.63, −0.20], *p* < 0.001	Chi^2^ = 19.65, *p* = 0.19, *I*^2^ = 24%
Fasting glucose (mmol/L)	16	−0.2 [−0.4, 0.1], *p* = 0.14	Chi^2^ = 14.12, *p* = 0.52, *I*^2^ = 0%

*Min, minutes; MAGE, mean amplitude of glycemic excursions; hyperglycemia, typically defined as >10 mmol/L; hypoglycemia, typically defined as <3.9 mmol/L*.

### Effect of Longer-Term (>2 Weeks) Exercise Training on Glucose Concentrations

**Figure 4:**
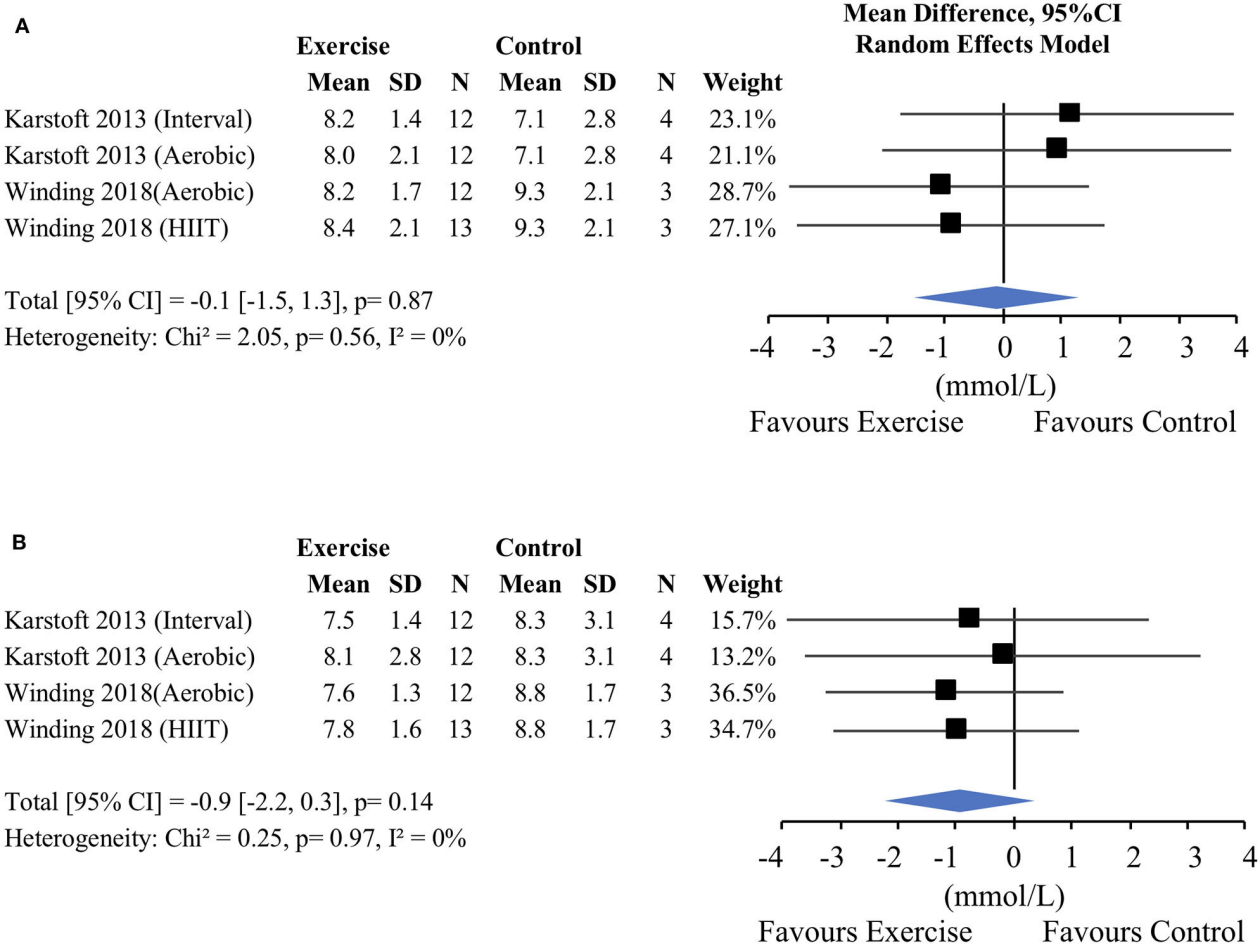
Mean 24-h glucose concentrations in longer-term (>2 weeks) studies. **(A)** Exercise vs. control pre-intervention, **(B)** exercise vs. control post-intervention. CI, confidence interval; SE, standard error; 1RM, one repetition maximum; HIIT, high-intensity interval training.

Four of the studies started post-training CGM measures 48–72 h after the last bout of exercise and described the post-intervention measurements within 1 week of the last bout of exercise. There was no baseline difference in mean 24-h glucose concentrations between exercise and non-exercise control groups (−0.1 mmol/L [−1.5, 1.3], *p* = 0.87, *I*^2^ = 0%; Figure 4A). When the post-intervention results were pooled, mean 24-h glucose concentration was not significantly lower in the exercise groups compared with the control groups (−0.9 mmol/L [−2.2, 0.3] *p* = 0.14, *I*^2^ = 0%; Figure 4B). However, only 4 exercise conditions were included in this exercise vs. control comparison with a total of 49 participants in the exercise groups and 15 in the control groups.

**Figure 5:**
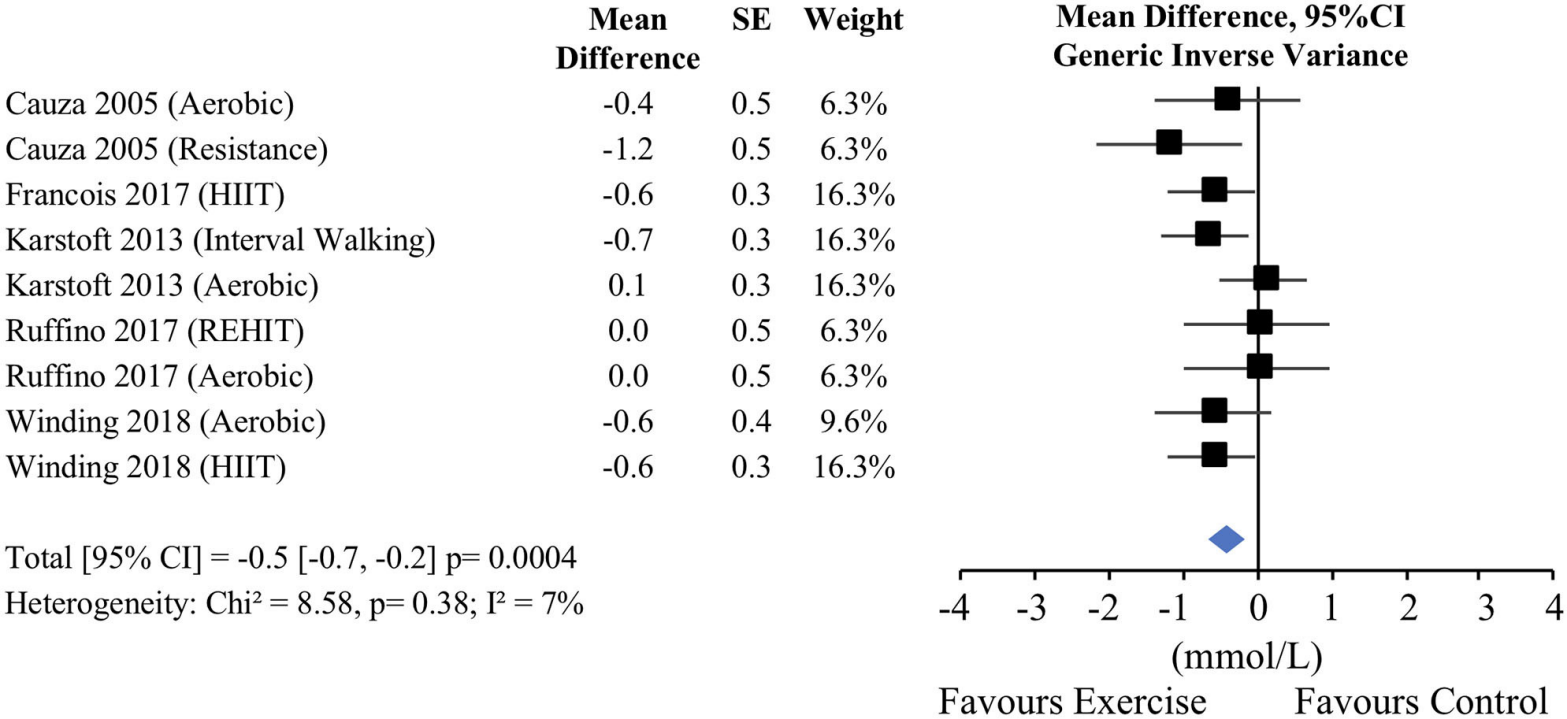
Mean 24-h glucose concentrations in longer-term (>2 weeks) studies pre- vs. post-exercise. CI, confidence interval; SE, standard error; 1RM, one repetition maximum; HIIT, high-intensity interval training; REHIT, reduced exertion high intensity interval training.

Secondary analysis of the pre- and post-exercise comparisons resulted in the inclusion of 9 longer-term exercise conditions with a total of 115 participants. Compared to pre-exercise values, post-exercise 24-h mean glucose concentrations significantly decreased (−0.5 mmol/L [−0.7, −0.2], *p* < 0.0002, *I*^2^ = 1%; Figure 5). Subgroup analyses, regression analyses, and examination of other outcomes were not performed due to the low number of available comparisons.

### Risk of Bias

Summaries according to the Cochrane Collaboration Risk of Bias tool are provided in Supplementary Figures 1, 2 for short and longer-term studies, respectively. Most of the included studies described their intervention as randomized but did not describe the methods of randomization, resulting in the categorization of “unknown” risk of bias on this criterion. Some of the trials that were described as randomized trials were actually categorized as “high” risk of bias because the randomization only affected the multiple exercise conditions and control condition always took place before exercise. When we performed subgroup analyses among the short-term studies to compare the “low” or “unknown” to “high” risk of biases on the random sequence generation criteria there was no difference between these types of studies on 24-h glucose concentrations (−0.5 mmol/L [−0.6, −0.3] vs. −0.4 mmol/L [−0.9, 0.1], respectively; see Table 3). As expected in exercise trials, blinding of participants to the exercise intervention is not feasible.

Funnel plots were also generated to examine the potential for publication bias. For the primary outcome of mean 24-h glucose concentrations, funnel plots are provided in Supplementary Figures 3, 4 for short and longer-term studies, respectively. Visual inspection of the funnel plots did not reveal any asymmetries, with the exception of the outlier from Cruz et al. (40) which found a comparatively large 2.8 mmol/L decrease in one of their short-term exercise groups. However, this group also had average size SE, which would not be expected in a typical publication bias scenario where studies with the largest SE tend to show more beneficial effects.

## Discussion

The present systematic review and meta-analyses confirms our previous findings that exercise reduces mean 24-h glucose and time spent in hyperglycemia (5), but also builds on this 2013 work in several ways:

1. The number of eligible short-term studies reporting the effects of exercise on CGM outcomes in T2D has approximately tripled (from 8 to 23 studies; or from 116 to 373 participants).
2. The greater number of short-term studies allowed for hypothesis generating subgroup and meta-regression analyses, which helped explain the heterogeneous responses among trials (e.g., the effects of exercise timing, sex, and glycemic control).
3. There were a sufficient number of trials to include outcomes that were not previously considered; including glycemic variability in short-term studies and mean 24-h glucose in longer-term studies.

The improvement in mean 24-h glucose concentrations following short-term exercise was 0.8 mmol/L in our 2013 meta-analyses and 0.5 mmol/L in the current one. These means were outside of each other's 95% confidence intervals. The differences may be due to the higher variability among trials in our current review as reflected in the higher *I*^2^-value (i.e., 3 vs. 73%) and the addition of recent studies in which glucose concentrations were unchanged following exercise [e.g., Rees et al. (9)].

As in our previous meta-analysis (5), exercise did not affect fasting glucose (−0.2 mmol/L [−0.4, 0.1], *p* = 0.14). It is possible that this would have reached statistical significance had fasting glucose been reported in more short-term studies. Nonetheless, it may be that exercise has a greater impact on postprandial glucose, which is more strongly linked to muscle insulin resistance, whereas fasting glucose is believed to be more strongly associated with hepatic insulin resistance (63, 64). Longer-term studies have shown reductions in fasting glucose with exercise (65), but it is difficult to know to what extent this was due to weight loss.

To better understand the heterogeneity among short-term trials, we conducted a series of subgroup meta-regression analyses. It is important to note that since participants were not randomly assigned to the subgroups, we cannot determine if it was a causal relationship. In addition, some variables in our subgroup and meta-regression analyses were not pre-specified. Consequently, results from our subgroup analyses should be interpreted with caution and confirmed by randomized trials. In our meta-regression analyses, the strongest predictor of greater improvements in glycemic control was the mean 24-h glucose concentrations from the control condition, suggesting that participants with elevated glucose concentrations had greater reductions following exercise. Although this may seem intuitive, it is potentially affected by a regression to the mean artifact [as previously reviewed by Sheppard (66)]. However, the association between baseline A1C and changes in mean 24-h glucose following exercise was in the same direction (*r* = −0.33, *p* = 0.03). Sex, but not age or BMI, was associated with changes in mean 24-h glucose. Studies that had a higher proportion of males were associated with greater reductions in mean 24-h glucose. Our meta-analysis does not permit us to identify the reasons why males may have responded more favorably compared to females. However, a greater effect of exercise on insulin sensitivity (67) and post-exercise glucose metabolism (68) has been previously observed in males compared to females. The reasons for these differences are not well-known, but may be related to differences in substrate oxidation during exercise and recovery (69). Of note, only 3 women were not postmenopausal among the 6 studies that reported menopausal status. Consequently, it is possible that the results are not generalizable to women before menopause.

However, we cannot rule out that the association with sex was caused by other confounders and we noted very high heterogeneity among the studies that only included males (see left side of Figure 3B). The association observed between the proportion of females and changes in mean 24-h glucose following exercise was only observed after removing of a potential outlier. Indeed, the study by Cruz et al. (40) was the only study that included only female participants (*n* = 12). They compared a single bout of exercise performed at 80 vs. 40% of the participants individualized one-repetition maximum (i.e., the heaviest weight that could be lifted once for each of 7 exercises). Resistance training was performed with a circuit in which each exercise was performed 3 times. While exercise at 80% increased mean 24-h glucose by 0.2 mmol/L, exercise at 40% reduced it by 2.8 mmol/L. To put this in perspective, this reduction is more than 5 times as much as the mean reduction in our meta-analyses and nearly twice as much as the next largest reduction among the 39 exercise conditions. The authors suggest that the greater counterregulatory hormone responses with the greater resistance exercise intensity may have contributed to the differences between conditions. It is also noteworthy that the participants in the Cruz et al. study were also the ones with the highest mean 24-h glucose during the control condition and therefore had the potential for greater reductions without experiencing hypoglycemia.

The timing of exercise was associated with some of the variance among short-term studies. Again, in our subgroup analyses, most participants were not randomly assigned to different exercise timing and therefore causality cannot be inferred. However, five studies directly compared two similar amounts of exercise performed at different times of the day (39, 43, 54, 56, 70). The results from Savikj et al. (39) contradict the findings from our meta-analyses and suggest exercise performed in the morning was less effective than afternoon exercise. However, this study involved HIIT training whereas most of the studies in our meta-analyses did not. They also offered a snack after morning exercise only. If changes in the timing of exercise can be found to consistently affect glycemic responses, this could be encouraging for people with T2D who could use such strategies to get more benefits from the same amount of exercise. The decision to perform subgroup analyses based on exercise timing in relation to meals was *a priori* as a consequence of our findings in the study by Rees et al. (9), which used afternoon exercise. However, we were unsure of the exact subgroups that would be available (e.g., we expected to have evening/post-dinner exercise subgroups?) and divided our subgroups in a way to have multiple studies in each subgroup.

The reasons why fasting (i.e., before breakfast) exercise would lead to significant and consistent reductions in mean 24-h glucose, while afternoon exercise did not, are not well-understood. One potential explanation could be that, in the absence of exogenous fuels, fasting exercise must rely to a greater extent on endogenous fuels (e.g., intramuscular lipids and glycogen) and that these changes may favor an increase in insulin sensitivity. The first two longer-term training studies comparing fasting exercise to postprandial exercise in T2D have been recently published (71, 72). These longer-terms studies did not support a more favorable effect of fasting exercise compared to postprandial exercise. However, the postprandial exercise was performed shortly after breakfast (not in the afternoon) in both of these studies (71, 72). It is currently difficult to understand to what extent the effects of fasting exercise are due to fasting itself or to the time of day (i.e., diurnal variations). To further complicate matters, in people with T2D, many glucose lowering medications are taken with meals and we found an association with the use of sulfonylurea within a study in changes in 24-hr glucose following exercise vs. control, but not for other categories of medication.

Interpretation of differences among subgroups is based on comparing results from different exercise conditions that did not benefit from randomization, therefore subgroup comparisons may be affected by several confounding variables and should be confirmed by randomized trials. Several studies included in our meta-analysis did directly compare the effect of different exercise intensities. Some compared continuous exercise to different forms of higher intensity interval training (45, 49, 54, 73), one compared low vs. moderate intensity continuous exercise (48), and one compared different intensities of resistance exercise (40). As in the subgroup analyses from our meta-analysis, no clear pattern emerged when examining these studies individually. However, a previous meta-analysis of longer-term studies with head-to-head comparison of exercise of different intensities suggested that higher intensity exercise led to greater declines A1C (8). Another difference was that the trials in the earlier meta-analysis had similar or greater energy expenditures in the high intensity groups compared to the lower intensity groups from the same trial. Likewise, the aerobic vs. resistance training comparison in the short-term trials may not reflect longer term adaptations. The mechanisms leading to improvements in glycemic control following continuous aerobic, HIIT and resistance training may be different, and are beyond the scope of our meta-analysis.

Methodological aspects unrelated to exercise, such as the type of CGM (real-time vs. blinded vs. intermittently scanned) as well as the level of dietary control (i.e., the provision of meals), did not significantly explain the heterogeneity among trials in regards to changes in mean 24-h glucose. However, the absence of significant subgroup differences may be due to the presence of other confounders as there was high heterogeneity within many different subgroups. The type of CGM or the degree of dietary control may influence compensatory behaviors from participants (e.g., eating more if glucose is known to be low).

Glycemic variability may be independently associated with cardiovascular disease (74). When examining the change across all short-term studies, we observed a consistent and statistically significant reduction in MAGE. However, within each individual study the 95% confidence interval would often overlap with zero, suggesting that individual studies were often underpowered to detect differences. There were several indices of glycemic variability. Although these indices differ in their calculations, they were highly related to each other. For example, correlation coefficients were all above 0.85 among MAGE, CONGA, and SD (75).

There were fewer longer-term studies identified and only two with randomization to a non-exercise control condition. The pre- vs. post-analyses led to different conclusions than the randomized exercise vs. control comparison. The pre- vs. post-comparison had a smaller mean difference but reached statistical significance, in part due to the greater number of participants but also because of the increased statistical power within participant analyses. Interestingly, the weighted mean difference in the pre- and post-analyses was similar to the weighted mean difference found in the acute studies (i.e., 0.5 mmol/L). Based on conversions between A1C and estimated average glucose (76), such a reductions could correspond to a 0.3 percentage point reduction in A1C, which is lower than previous meta-analyses of exercise trials with A1C as a primary outcome (1, 2). This is not surprising given that the post-training CGM measures typically started at least 48-h after the last bout of exercise to minimize the acute effect from this last bout. Therefore, we would expect the weekly average glucose to be lower in these participants who prescribed exercise three times per week or more. Weight loss in longer-term exercise trials may mediate some of the improvements in glycemic control. For eight of the nine longer-term exercise conditions, changes in body weight were ≤1 kg. Consequently, we believe that most of the changes were observed in the absence of meaningful weight loss.

The main limitation of this meta-analysis is the high heterogeneity among the shorter-term studies and that we were only partially successful at explaining the heterogeneity. Consequently, interpreting the overall effects should be done with caution. Based on our findings, it is unlikely that exercise increases blood glucose; it is more likely that the heterogeneity is in the degree of the positive to no effects. The apparent heterogeneity may in fact be in part a result of the analytical approach that we chose. Indeed, the within participant mean change and SE used in the generic inverse method approach, leads to much narrower confidence internals than if we compared the mean glucose from the exercise vs. control using the between participant standard deviation in each condition. When the latter approach is used, the weighted mean difference remained similar (0.4 mmol/L [−0.70, −0.20]) but the heterogeneity is greatly reduced (Chi^2^ = 24.5, *p* = 0.96), *I*^2^ = 0%) since the mean difference found in each study has wider confidence intervals. The heterogeneity may also be caused my methodological issues. Several CGM devices require multiple calibrations per day and errors in calibration values can have a meaningful impact on 24-h outcomes. In addition, investigators often have to make difficult decisions on how to treat missing CGM values. Lastly, another limitation is the low number of longer-term studies and we would caution against inferring that chronic exercise training no more effective than shorter-term exercise due to the timing of the CGM measures in the longer-term studies.

In conclusion, both short-term and long-term exercise can reduce mean 24-h glucose concentrations. Short-term exercise also reduces other CGM-derived outcomes such as glycemic variability, while additional longer-term studies are needed to examine such outcomes. The glycemic response to short-term exercise can be variable, and exploratory analyses suggests that the heterogeneity among studies might in part be explained by the extent to which glycaemia is impaired on non-exercise days, or factors such as the timing of exercise and the sex of participants.

## Data Availability Statement

The original contributions presented in the study are included in the article/Supplementary Material, further inquiries can be directed to the corresponding author/s.

## Author Contributions

MM, CO, AM-C, and JR contributed to data extraction. MM, CO, and NB performed the statistical analysis and wrote sections of the manuscript. All authors contributed to the conception and design of the study, manuscript revision, read, and approved the submitted version.

## Conflict of Interest

NB has received continuous glucose monitors from Medtronic Canada for previous studies. The remaining authors declare that the research was conducted in the absence of any commercial or financial relationships that could be construed as a potential conflict of interest.
