# Supplementary material for: Acute and Chronic Effects of Exercise on Continuous Glucose Monitoring Outcomes in Type 2 Diabetes: A Meta-Analysis
Source: Front Endocrinol (Lausanne). 2020 Aug 4;11:495. doi: 10.3389/fendo.2020.00495 (PMC7417355; doi:10.3389/fendo.2020.00495)

Supplementary Material.

Supplementary table 1. Literature search strategy **(searched on January 9, 2020)**

	Intervention terms		Population terms		Outcome terms	Hits
PubMed	Exercise (MeSH) OR Motor Activity (MeSH) OR exercise OR sports OR physical education OR exertion OR motor activity OR motor activity OR exercise* OR "physical activity" OR "physically active" OR train* OR fitness OR aerobic* OR sport OR sports OR running OR jog OR jogging OR swim* OR cycling OR bicycle* OR walk* OR "weight lifting" OR "weight training" OR "resistance training"	AND	Type 2 Diabetes (MeSH) OR type 2 diabetes OR niddm OR non insulin dependent diabetes OR adult onset diabetes OR diabetes mellitus type ii	AND	continuous glucose monitor* OR continuous subcutaneous glucose monitor* OR cgm OR cgms OR continuous glucose monitoring OR Flash glucose monitor*	212
Medline[1]	exp. Exercise OR exp. Motor Activity OR exercise or sports or physical education or exertion or motor activity or motor activity or exercise* or "physical activity" or "physically active" or train* or fitness or aerobic* or sport or sports or running or jog or jogging or swim* or cycling or bicycle* or walk* or "weight lifting" or "weight training" or "resistance training".mp	AND	exp. Diabetes Mellitus, Type 2 or type 2 diabetes or niddm OR non insulin dependent diabetes or adult onset diabetes or diabetes mellitus type ii.mp	AND	continuous glucose monitor* or continuous subcutaneous glucose monitor* or cgm or cgms OR continuous glucose monitoring or Flash glucose monitor*	129
Embase[2]	exp Exercise OR Exp Motor Activity OR exercise or sports or physical education or exertion or motor activity or motor activity or exercise* or "physical activity" or "physically active" or train* or fitness or aerobic* or sport or sports or running or jog or jogging or swim* or cycling or bicycle* or walk* or "weight lifting" or "weight training" or "resistance training".mp	AND	exp. Diabetes Mellitus, Type 2 or type 2 diabetes or niddm OR non insulin dependent diabetes or adult onset diabetes or diabetes mellitus type ii.mp	AND	continuous glucose monitor* or continuous subcutaneous glucose monitor* or cgm or cgms OR continuous glucose monitoring or Flash glucose monitor*	316

[1] Ovid MEDLINE(R) and Epub Ahead of Print, In-process & other non-indexed citations and daily 1946 to January 08, 2020
[2] Embase 1974 to 2020 January 8

Supplementary Figure 1. Risk of bias for short-term (≤2 weeks) studies (n=23)

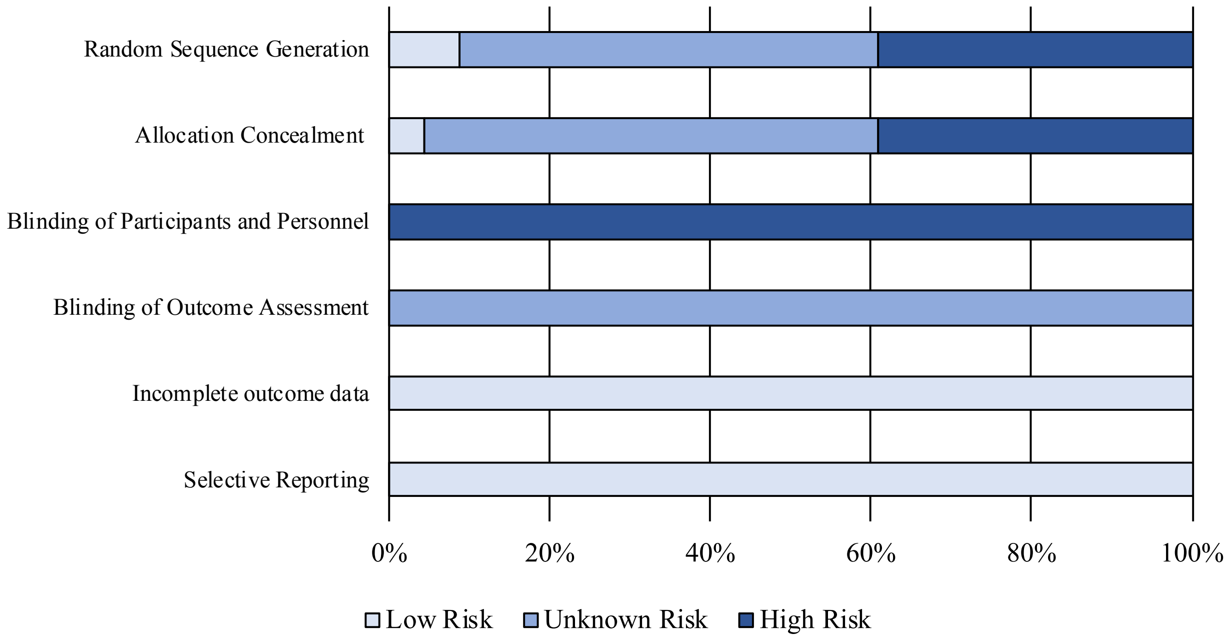

Supplementary Figure 2. Risk of bias for longer-term (>2 weeks) studies (n=5)

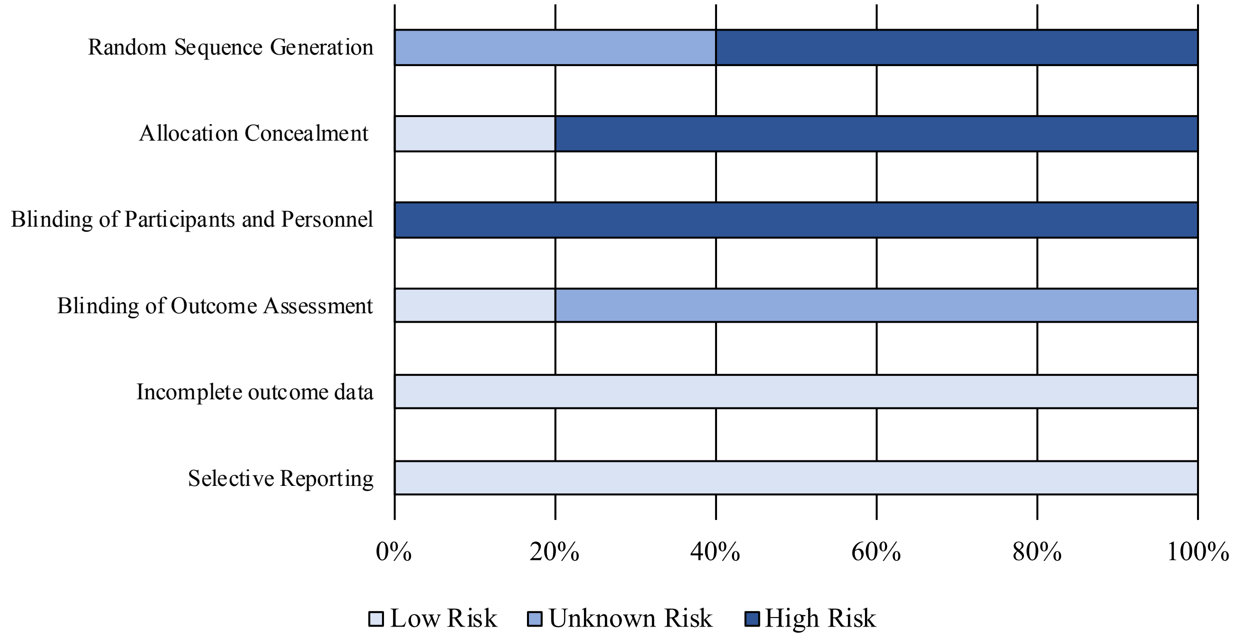

Supplementary Figure 3. Funnel plot for mean 24-hour glucose in short-term (□2 weeks) studies.

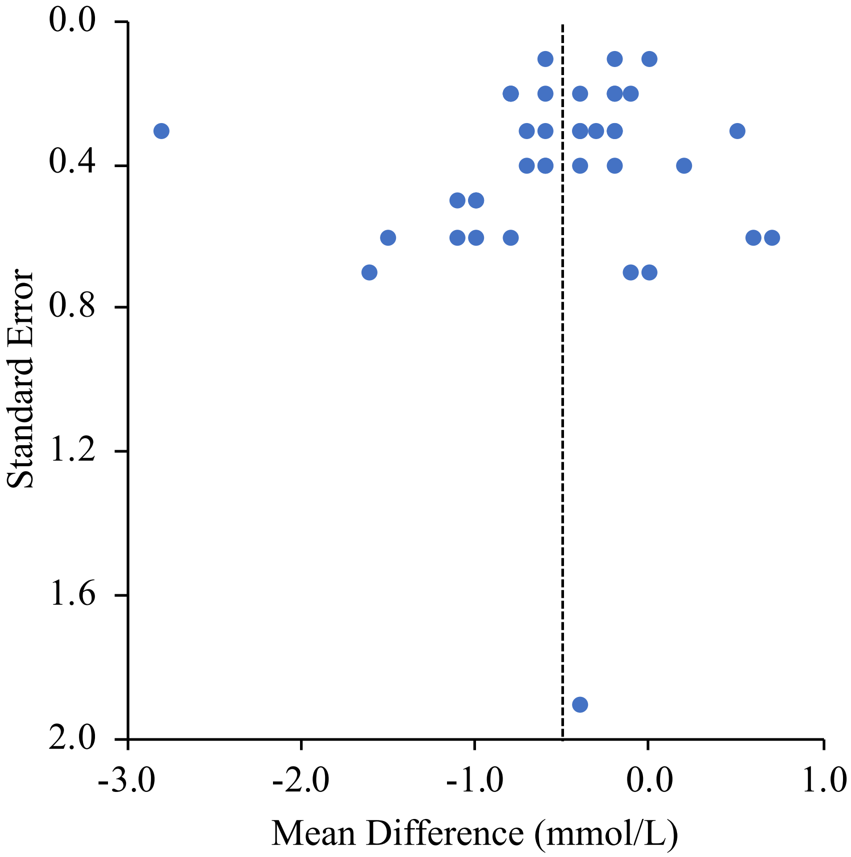

Supplementary Figure 4. Funnel plot for mean 24-hour glucose in long-term (>2 weeks) studies. A) Post intervention exercise vs control analysis, B) Pre vs post exercise analysis.

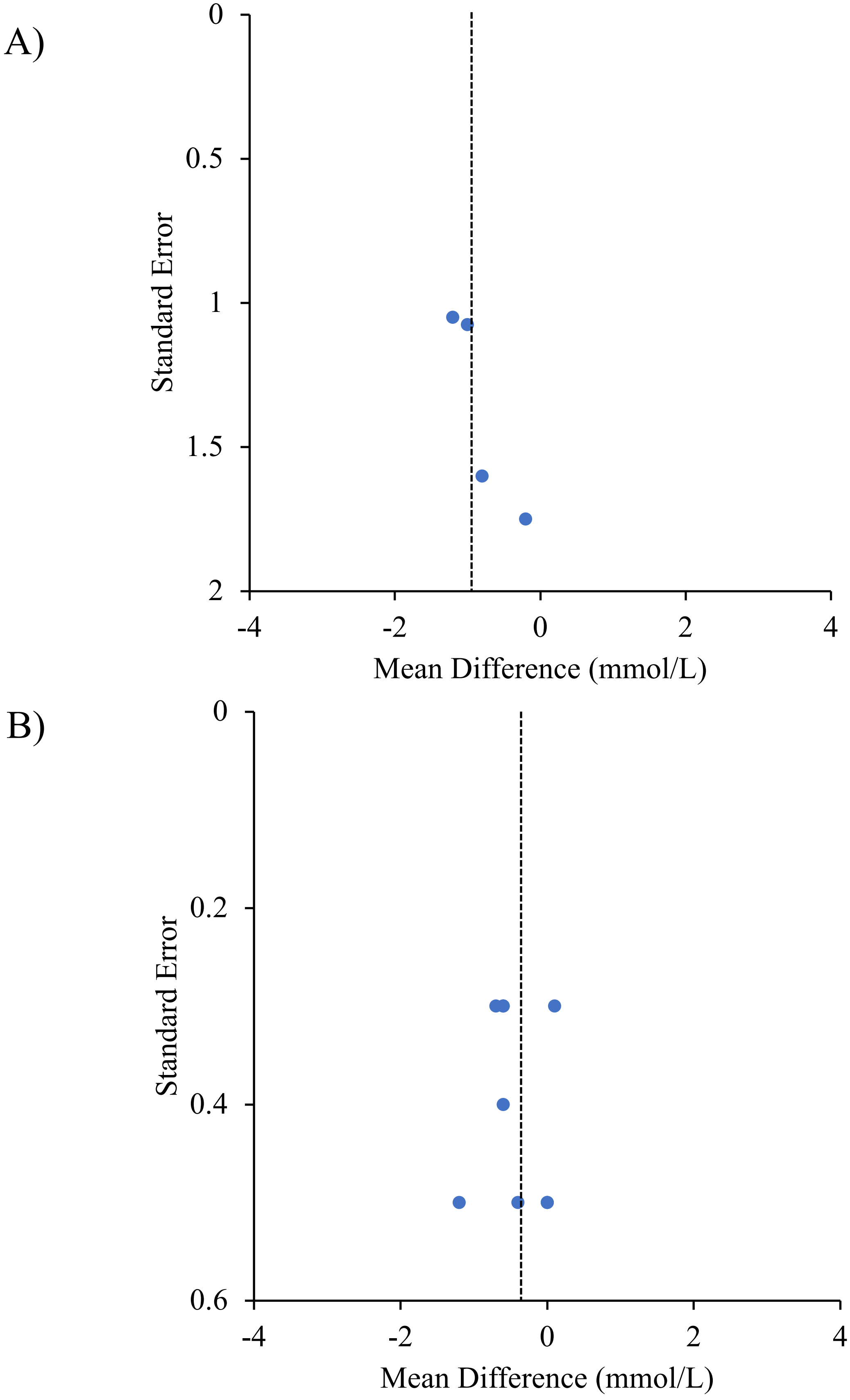

Supplement: Supplementary file 1 [file Data_Sheet_1.PDF]
